# NDR kinase *tricornered* genetically interacts with *Ccm3* and metabolic enzymes in *Drosophila melanogaster* tracheal development

**DOI:** 10.1093/g3journal/jkad013

**Published:** 2023-01-19

**Authors:** Joshua Hudson, Sayantanee Paul, Alexey Veraksa, Amin Ghabrial, Kieran F Harvey, Carole Poon

**Affiliations:** Peter MacCallum Cancer Centre, 305 Grattan Street, Melbourne, Victoria 3000, Australia; Sir Peter MacCallum Department of Oncology, The University of Melbourne, Parkville, Victoria 3010, Australia; Department of Biology, University of Massachusetts Boston, Boston, MA 02125, USA; Department of Biology, University of Massachusetts Boston, Boston, MA 02125, USA; Department of Pathology and Cell Biology, Columbia University College of Physicians and Surgeons, 630 West 168th Street, New York, NY 10032, USA; Peter MacCallum Cancer Centre, 305 Grattan Street, Melbourne, Victoria 3000, Australia; Sir Peter MacCallum Department of Oncology, The University of Melbourne, Parkville, Victoria 3010, Australia; Department of Anatomy and Developmental Biology, and Biomedicine Discovery Institute, Monash University, Clayton 3800, Australia; Peter MacCallum Cancer Centre, 305 Grattan Street, Melbourne, Victoria 3000, Australia; Sir Peter MacCallum Department of Oncology, The University of Melbourne, Parkville, Victoria 3010, Australia

**Keywords:** NDR, hippo-like, trachea, CCM3, metabolism

## Abstract

The Germinal Center Kinase III (GckIII) pathway is a Hippo-like kinase module defined by sequential activation of Ste20 kinases Thousand and One (Tao) and GckIII, followed by nuclear dbf2-related (NDR) kinase Tricornered (Trc). We previously uncovered a role for the GckIII pathway in *Drosophila melanogaster* tracheal (respiratory) tube morphology. The trachea form a network of branched epithelial tubes essential for oxygen transport, and are structurally analogous to branched tubular organs in vertebrates, such as the vascular system. In the absence of GckIII pathway function, aberrant dilations form in tracheal tubes characterized by mislocalized junctional and apical proteins, suggesting that the pathway is important in maintaining tube integrity in development. Here, we observed a genetic interaction between *trc* and *Cerebral cavernous malformations 3* (*Ccm3),* the *Drosophila* ortholog of a human vascular disease gene, supporting our hypothesis that the GckIII pathway functions downstream of Ccm3 in trachea, and potentially in the vertebrate cerebral vasculature. However, how GckIII pathway signaling is regulated and the mechanisms that underpin its function in tracheal development are unknown. We undertook biochemical and genetic approaches to identify proteins that interact with Trc, the most downstream GckIII pathway kinase. We found that known GckIII and NDR scaffold proteins are likely to control GckIII pathway signaling in tracheal development, consistent with their conserved roles in Hippo-like modules. Furthermore, we show genetic interactions between *trc* and multiple enzymes in glycolysis and oxidative phosphorylation, suggesting a potential function of the GckIII pathway in integrating cellular energy requirements with maintenance of tube integrity.

## Introduction

The *Drosophila melanogaster* tracheal (respiratory) system is a branched, three-dimensional network of epithelial tubes essential for oxygen transport (Schottenfeld et al. 2010). These specialized tubes form in a stereotypic pattern during embryogenesis in response to intrinsic and extrinsic cues and are analogous in cellular organization to branched tubular structures in vertebrates such as the lung, kidney and vasculature (Ochoa-espinosa *et al.* 2012). In the fly, the tracheal network is essential for transporting gasses throughout the body via tubules of three distinct architectures that become progressively smaller in diameter as they extend from a pair of dorsomedial multicellular tubes to single-celled tubes ramifying on target sites (Ghabrial *et al.* 2003; Hayashi and Dong 2017). These fine tubules form the majority of the tracheal network and therefore maintenance of correct tube architecture is critical, requiring precise coordination of junctional remodeling within cells and between adjoining cells to preserve an intact lumen.

Early in development, hardwired genetic programming largely determines the architecture of embryonic tracheal system. During larval stages, the most well established driver of tracheal plasticity in *Drosophila* is oxygen availability (Jarecki *et al*.1999; Centanin *et al.* 2008), where both the number of branches and their length and diameter are controlled by integrating developmental signals with changes in gas requirements (Uv *et al.* 2003). Furthermore, alterations in oxygen levels or nutrition in larvae can also affect insulin signaling to control the number of tracheal branches and overall larval growth (Linneweber *et al.* 2014; Wong *et al.* 2014; Burguete *et al.* 2019; Yuan *et al.* 2020). In addition to these systemic responses to oxygen and insulin signaling, many cellular processes are important in shaping the fine terminal cell branches themselves. Morphogenesis occurs in the absence of cell division, relying on cell rearrangement and remodeling of cell junctions to shape branch morphology while maintaining tube integrity (Ghabrial *et al.* 2003; Hayashi and Dong 2017). This includes microtubule and actin cytoskeleton dynamics, which support outgrowth of terminal branches, and vesicle trafficking mediated by dynein or endocytosis, which supports the formation and elongation of the apical lumens of tubes (Schottenfeld-Roames and Ghabrial 2012; Schottenfeld-Roames *et al*. 2014; Hayashi and Kondo 2018; Mathew *et al.* 2020; Ricolo and Araujo 2020; Rios-Barrera and Leptin 2022). Here, we propose that terminal cell tube morphology may also be responsive to changes in metabolic pathways, including glycolysis and oxidative phosphorylation, in coordination with tricornered (Trc) kinase, a downstream kinase of the GckIII pathway.

**Fig. 1.**
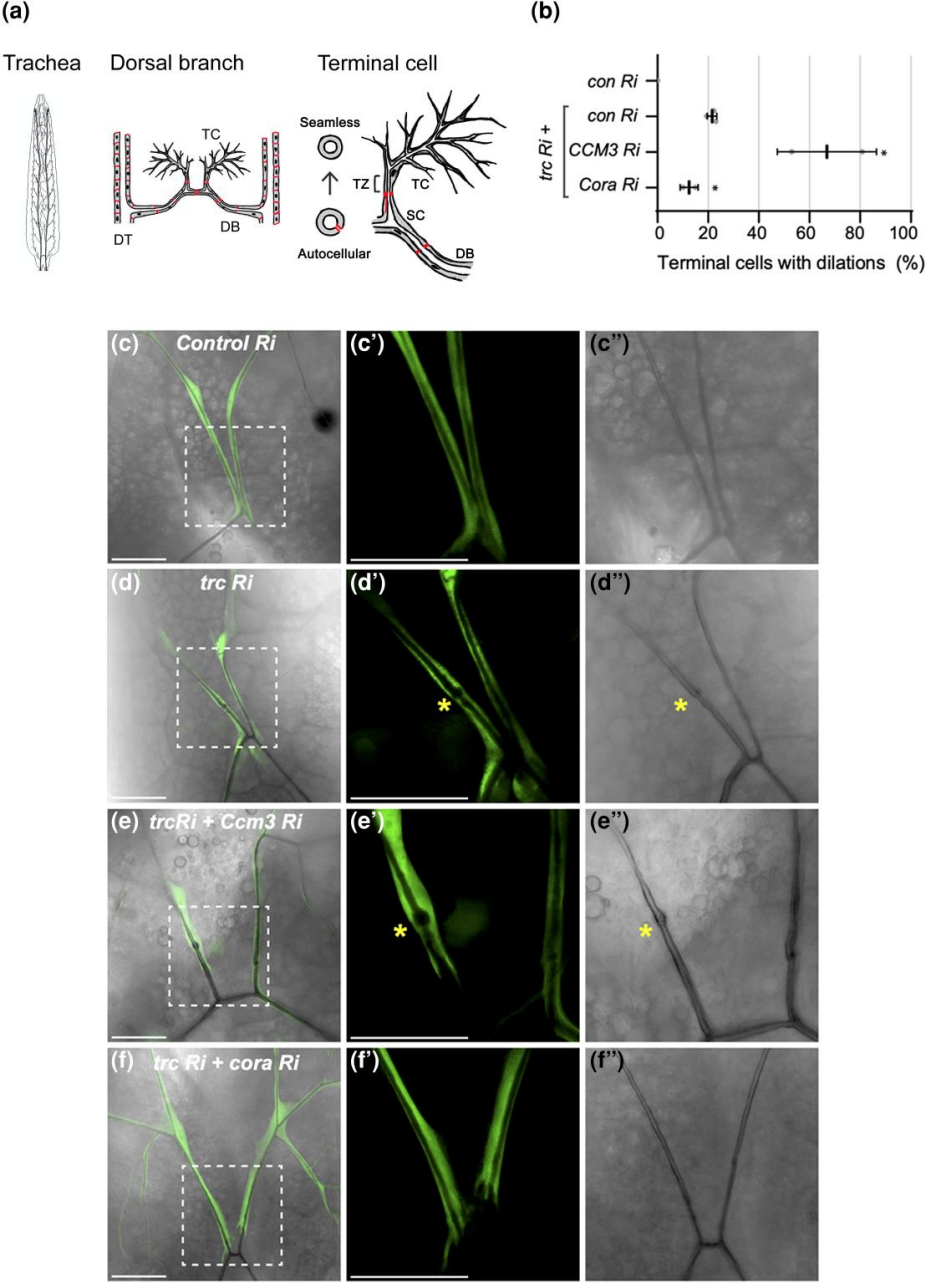
*trc* genetically interacts with *Ccm3* and *Cora* in trachea terminal cells. a) Schematic diagram of the *Drosophila* trachea system in third instar larvae (dorsal view, anterior up). A pair of multicellular dorsal trunks (DT) span the anterior-posterior axis between which 10 dorsal branches (DBs), composed of autocellular tubes, laterally connect. From each dorsal branch (DB), terminal cell (TC) pairs extend anteriorly. Terminal cells are composed of seamless tubes, and connect to the DB via the stalk cell (SC), an autocellular tube. The transition zone (TZ) is a critical region where autocellular tubes connect to seamless tubes and is located between the intercellular junction of the SC and the nucleus/cell body of the terminal cell (marked by square bracket). Screen phenotypes were assessed in the transition zone. b) Quantification of the number of terminal cells with dilations. Indicated RNAi lines are expressed by *drm > GFP* and shown in C–F”. Bars represent mean + SEM. Genotypes and number of terminal cells assessed (*n* value): *trc Ri* + *control* (luciferase) *Ri, n* = 27, 36, 26; *trc Ri* + *CCM3 Ri, n* = 23,23; *trc Ri* + *Cora Ri, n* = 20, 19. Bars represent mean +/− SEM. An unpaired two-sided Student's t-test was used to determine statistical significance between *trc Ri* + *con Ri* control and *trc Ri* + *additional RNAi* experimental genotypes * *P*-value < 0.05. (C–F’’) Confocal images of terminal cells from third instar larvae (dorsal view) expressing *drm > GFP* (fluorescent signal). Bright field shows lumen outline. Dashed square box indicates region that is zoomed in on the two right panels. Scale bars, 50 µM. (C–C’). Terminal cells expressing GFP and a control *luciferase RNAi* have smooth continuous lumens. (D–D’) Expression of *trc RNAi* results in 21% terminal cells harboring dilations in the transition zone (asterisk). (E–E’) Co-expression of *trc RNAi* with *CCM3 RNAi* results in enhanced dilation rate in terminal cell transition zone (asterisk) compared with *trc RNAi* alone (B). f) Co-expression of *trc RNAi* with *Coracle (Cora) RNAi* results in a partial suppression of the dilation rate of *trc RNAi* alone (B).

We recently demonstrated that the GckIII pathway is a Hippo-like kinase cascade utilized by terminal cells to preserve tube architecture in development. Hippo-like kinase modules are highly conserved from fungi to vertebrates in organization and function, controlling processes such as cell division and morphogenesis. These modules are characterized by the sequential activation of an upstream activator protein, an MST kinase, and an NDR kinase, which finally regulates target effector proteins (Thompson and Sahai 2015). The GckIII pathway is comprised of the Ste20 kinases Thousand and one amino acid (Tao) and Germinal center kinase III (GckIII), and the nuclear dbf1-related (NDR) kinase Trc, orthologous to vertebrate Stk38, 38L. Upon loss of any one of the GckIII pathway kinases, dilations were observed specifically in the transition zone of larval terminal cells, concomitant with mislocalized junctional and apical proteins (Song *et al.*2013; Poon *et al.* 2018). The transition zone is a specific region of the terminal cell where autocellular tubes connect to seamless tubes and is located between the intercellular junction of the terminal cell with its neighboring stalk cell, and the terminal cell nucleus, generally preceding ramification of the terminal cell branches (marked by bracket in Fig. 1a) (Samakovlis *et al.* 1996). In configuring seamless tube structure, it is thought that the septate junctions which zip up one side of the autocellular tube are remodeled away to form a seamless tube with a continuous apical membrane facing the lumenal space (Song *et al*. 2013). In the absence of GckIII pathway function, aberrant dilations predominate in the terminal cell transition zone concomitant with mislocalized septate junction proteins, such as Fasiclin3 (Fas3) and Coracle (Cora) (Song *et al*. 2013; Poon *et al.* 2018), as well as an enrichment of Rab11 (Song *et al*. 2013). However, many of the mechanisms by which the GckIII pathway regulates tracheal development are currently unknown.

Another conserved feature of Hippo-like kinase pathways is the requirement for specific scaffold proteins. These regulate the activity and localization of kinases, such as Mouse protein 25 (Mo25) that regulates GckIII kinases, or Monopolar spindle one binder (Mob) family and Furry (Fry) proteins, that regulate NDR kinases (Hergovich *et al.* 2006; Mehellou *et al.* 2013; Sugden *et al*. 2013; Gundogdu and Hergovich 2019). Whether these scaffold proteins are required for the GckIII pathway in tracheal development has been unclear.

Familial forms of the human vascular disease, cerebral cavernous malformation (CCM), arises from mutation in any of three genes (CCM1/KRIT1, CCM2/Malcavernin, and CCM3/PDCD10). Human GckIII kinases (STK24, STK25, and STK26) are established effectors of CCM3 (Ma *et al.* 2007; Voss *et al.* 2007). CCM vascular lesions are characterized by dilated and leaky cerebral capillaries and are associated with disrupted endothelial cell junctions that affect the integrity of the vessels (Fischer *et al.* 2013; Retta *et al*. 2020). To date, there are no direct therapies available for CCM disease, and the downstream signals that control how GckIII/CCM3 regulates vascular integrity are not well understood (Lant *et al.* 2015; Retta *et al.* 2020). The interaction between GckIII and CCM3 is conserved in multiple animal models, and in the fly, we have shown that loss of *Ccm3* enhances the *GckIII* mutant phenotype in trachea (Zheng *et al*. 2010; Yoruk *et al.* 2012; Song *et al.* 2013; Lant *et al*. 2015; Retta *et al.* 2020). Importantly, the fly offers a simplified system with single family members in the central kinase cascade (Tao, GckIII, and Trc), and single family members of most of the known scaffolding proteins (CCM3, Mo25, and Furry). Thus, *Drosophila* trachea can be utilized as a powerful model to investigate potential downstream targets of GckIII/CCM3 in vivo.

In prior work we demonstrated that Trc is a direct substrate of GckIII kinase activity, and that loss of *trc* function phenocopied *GckIII* tracheal defects in third instar larvae (Poone *et al*. 2018). Here, we show that *trc* genetically interacts with *Ccm3* in terminal cells, supporting our model that the Hippo-like GckIII-NDR kinase module is part of the downstream effector pathway of CCM3. To further investigate the mechanisms underpinning GckIII pathway signal transduction in tracheal development, we took biochemical and genetic approaches to discovering proteins that interact with Trc kinase, the most downstream kinase identified in the pathway. We tested Trc-interactors from the biochemical screens by RNAi-mediated knockdown, and identified a number that modified a *trc* loss of function phenotype in terminal cells. Among these interactors were established NDR kinase scaffold proteins, highlighting their conserved regulatory role in NDR kinase activity across different biological settings and organisms (Thompson and Sahai 2015). Additionally, we identified novel interactors that suggest cross-talk between NDR signaling and metabolic pathways during tracheal morphogenesis: knockdown of rate-limiting enzymes involved in glycolysis or oxidative phosphorylation enhanced the tube dilation defect associated with *trc* loss of function.

## Materials and methods

### Drosophila genetics

All stocks were maintained at 18°C on standard media, and crosses were cultured at 29°C unless otherwise indicated. Stocks used were: *drm-Gal4, UAS-GFP* (*drm >* GFP) (Green *et al.* 2002), *smg1^32AP^*; *btl-Gal4, UAS-Moesin::GFP* (*btl > MoeGFP, smg1^32AP^* is an allele of *smg1* [courtesy of M.Metzstein]; male progeny of crosses using virgins from this stock show enhanced expression of *UAS* constructs due to interference with nonsense mediated decay (Metzstein and Krasnow 2006). *btl > MoeGFP* crosses were incubated at 25°C and high GFP progeny were assessed), *UAS-trc RNAi* (41591, Bloomington), *UAS-luciferase RNAi* (31603, Bloomington), *UAS-Ccm3 RNAi* (109453, VDRC), *UAS-coracle RNAi* (51845, TRiP), *UAS-furry RNAi* (60103, Bloomington), *UAS-Mob2 RNAi* (107327, VDRC), *UAS-Mo25 RNAi* (55681, Bloomington), *UAS-PyK RNAi 1* and *2* (49533 & 35165, VDRC), *UAS-HexA RNAi 1* and *2* (104680, VDRC and 35155, Bloomington), *UAS-Pfk RNAi 1* and *2* (105666, VDRC and 36782, Bloomington), *UAS-blw RNAi* (28059, Bloomington), *UAS-ATPsyn-beta RNAi* (28056, Bloomington), and *UAS-ND75 RNAi 1* and *2* (33911 and 27739, Bloomington). Refer to Supplementary Table 2 and 3 for transgenic flies used in this study.

### Sensitized RNAi screen

Adult female flies of the genotype *drm*-*Gal4, UAS-trc RNAi* (41591, Bloomington) were crossed to adult male flies carrying UAS-RNAi targeting candidates to be tested for genetic interactions with *trc* (please refer to Supplementary Table 2 and 3 for transgenic flies screened), or to luciferase as a negative control (31603, Bloomington) to account for Gal4 dilution. Crosses were incubated at 29°C.

### Preparation and imaging of samples

Wandering third instar larvae of the correct genotype were heat fixed as described (Jones and Metzstein 2013). Briefly, larvae were collected in PBS, sorted for the correct genotype, and transferred to a drop of 100% glycerol on a glass slide. This slide was placed on a heat block at 70°C for 12–15 s to immobilize larvae. Larvae were oriented to the dorsal view, a coverslip was placed over the sample and sealed with nail polish. Imaging was performed within 3 h of heat immobilization on a Leica DMi8 inverted microscope (Columbia University Medical Center) or an Olympus FV3000 confocal laser scanning microscope (Peter MacCallum Cancer Centre). Images were processed using Leica LAS AF software (Leica), FIJI (Schindelin *et al.* 2012) and Adobe Photoshop (Adobe).

### Analysis of terminal cell phenotype

To assess terminal cell phenotypes, maximum intensity projection GFP images and the corresponding minimum intensity projection brightfield images of dorsal branch terminal cells were manually assessed and scored for dilation in the transition zone or other phenotypes in the terminal cells (refer to Fig. 1a, and Supplementary Tables 2 and 3 for results). Dilation rate was assessed as the percentage of terminal cell transition zones that had at least one dilation. If multiple dilations were observed in a terminal cell transition zone, it was not scored any differently to a terminal cell transition zone with a single dilation. Each data point graphed represents the mean dilation rate of the indicated genotypes.

### Mass spectrometry

Protein complexes containing Trc-GFP and associated interactors were purified from 0–16 hr embryos obtained from a cross between the ubiquitously expressed *da-GAL4* driver (55851, Bloomington) and *UAS-Trc-GFP* (32090, Bloomington) as in (Yang *et al.* 2016; Yang and Veraksa 2017) and analyzed by nanoLC-MS/MS. Results from two independent biological replicates were compared with two independent controls (embryos from *da-Gal4* crossed with *UAS-GFP*) using SAINT software (Choi *et al.* 2011) (Supplementary Table 1).

#### Statistical analysis

An unpaired two-sided Student's *t*-test, assuming equal standard deviation between sets of data, was used to determine statistical significance between the mean dilation rates of the candidate RNAi's with *trc* RNAi (where at least two replicate experiments were conducted) and *trc* RNAi and control luciferase RNAi (baseline dilation rate) using GraphPad Prism software (version 8.1.2). *P < 0.05, **P < 0.01, ***P < 0.001. *n* values are stated in figure legends and Supplemental Tables.

## Results

### *Trc* genetically interacts with *Ccm3* in tracheal development

The role of GckIII kinases as key effectors of CCM3 has important implications for human vascular disease, and the GckIII-CCM3 interaction is conserved in fly and vertebrate animal models (Ma *et al.* 2007; Fidalgo *et al.* 2010; Zheng *et al.* 2010; Chan *et al.* 2011; Song *et al.* 2013). Indeed, we previously showed that depletion of *Ccm3* in *Drosophila*, either using mutant alleles or via RNAi-mediated knockdown, enhances the *GckIII* loss of function tube dilation phenotype (Song *et al*. 2013). We previously described the GckIII pathway as a Hippo-like module, composed of Tao, GckIII and Trc that regulates tracheal morphogenesis in *Drosophila* (Poon *et al.* 2018). Because NDR kinases have yet to be directly implicated in CCMs, genetic interaction of *CCM3* and *trc* in *Drosophila* would be of particular interest, as it would suggest a central role of NDR kinase signaling in vascular biology and disease.

To assess the genetic interactions between the NDR kinase, *trc,* and other potential pathway components in tracheal development, we created a modifiable *trc* loss of function phenotype. Terminal cell expression of *trc* RNAi under control of *drm-GAL4,* along with GFP, to visualize the terminal cells (henceforth referred to as *drm > trc* RNAi), was combined with knockdown of luciferase (a negative control RNAi line, Fig. 1c), resulting in at least one dilation in the terminal cell transition zone in 21% of dorsal branch terminal cells (Fig. 1b and 1d).

To assess if *trc* genetically interacts with *Ccm3*, we co-expressed *Ccm3 RNAi* with *drm > trc* RNAi and observed that the frequency of dilations was enhanced to 67% of dorsal branch terminal cells (Fig. 1b and 1e). This is the first observation of a genetic interaction between *Ccm3* and *trc* in *Drosophila* trachea, and suggests the potential function of NDR kinase Trc downstream of CCM3 signaling as part of a Hippo-like GckIII-NDR kinase module.

### *Trc RNAi* dilation phenotype is suppressed by loss of septate junction protein Cora

We wished to demonstrate that the sensitized *trc* knockdown assay described above was also amenable to suppression. In the absence of GckIII pathway kinases, septate junction proteins, such as 4.1 protein Coracle (Cora), are mislocalized in terminal cell transition zone and terminal branches, contributing to the transition zone dilation defect (Song *et al*. 2013; Poon *et al.* 2018). Further, we showed that knockdown of the septate junction protein, Varicose, could suppress the *GckIII* loss of function phenotype (Song *et al*. 2013) and therefore predicted that knockdown of other septate junction proteins would suppress the *trc* RNAi phenotype. We found that knockdown of the septate junction protein, Cora, suppressed *the trc RNAi* dilation phenotype, resulting in 12.5% of terminal cells with dilations compared to the mean of 21% in *trc RNAi* terminal cells (Fig. 1b and 1f). These data suggest that reduction of septate junction proteins in general may suppress loss of function phenotypes of GckIII pathway kinases. Combined with the observation that loss of *Ccm3* enhanced the *drm > trc* RNAi phenotype, these data establish the *drm > trc* RNAi genetic interaction assay as one capable of identifying pathway components promoting or opposing signaling through the kinase module.

### Identifying Trc interacting proteins from biochemical screens

To further understand the role of *trc* in tracheal development, we tested knock down of candidate NDR kinase interactors identified either in this study in *Drosophila* or from published NDR kinase proteomic screens in vertebrates (Supplementary Table 1) (Ultanir *et al.* 2012; Gupta *et al.* 2013; Huttlin *et al.* 2015; Xiong *et al.* 2018), for their ability to modify the frequency of transition zone dilations in our sensitized *trc* RNAi background. We identified prospective Trc-binding proteins by performing protein affinity purification and mass spectrometry analysis of lysates from fly embryos expressing either Trc-GFP or GFP alone as a control (refer to Methods for details). The Trc-GFP fusion protein has not, to our knowledge, been tested for ability to rescue trc null mutants. Absent evidence for rescuing activity, it remains possible that the GFP-Trc fusion protein lacks essential Trc interactions. This biochemical screen detected peptides corresponding to known Trc-binding proteins, such as GckIII (Poon *et al.* 2018) and scaffold proteins Furry and Mob2 (Emoto *et al.* 2004; He *et al*. 2005b; Fang *et al.* 2010; Kohler *et al.* 2010) (Supplementary Table 1), suggesting a high likelihood that Trc-binding proteins could be detected in these experimental conditions.

### Sensitized RNAi screen to identify genetic interactions with *tr*c in tracheal development

RNAi lines targeting the top 10 proteins differentially identified in the Trc-GFP pulldown were selected (refer to Methods for details, Supplementary Table 2 and 3), together with RNAi lines targeting the *Drosophila* homologs of 11 candidates identified in NDR kinase proteomic screens in yeast or human cells (Ultanir *et al.* 2012; Gupta *et al.* 2013; Huttlin *et al.* 2015; Xiong *et al.* 2018) (refer to Supplemental Supplementary Table 2 and 3).

**Fig. 2.**
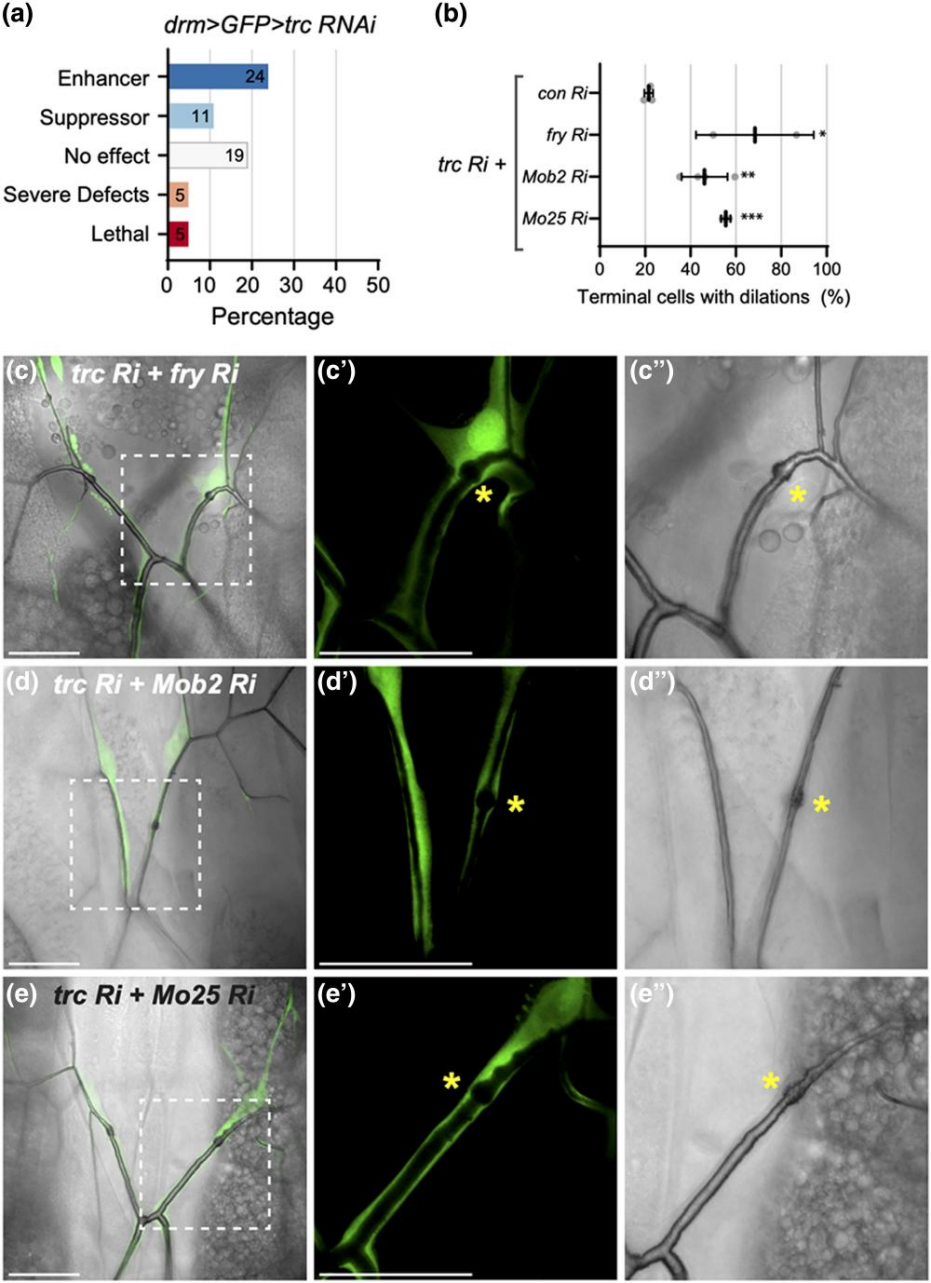
*trc* genetically interacts with GckIII and NDR scaffold proteins in trachea development. a) Summary of phenotypic categories identified in *drm > GFP > trc RNAi* genetic screen. b) Quantification of the number of terminal cells with dilations of the indicated genotypes shown in C–E’’). Mean +/− SEM shown. Genotypes and number of terminal cells assessed (*n* value): *trc Ri* + *control Ri, n* = 27, 36, 26; *trc Ri* + *fry Ri, n* = 17, 15; *trc Ri* + *Mob2 Ri, n* = 47, 37, 56; *trc Ri* + *Mo25 Ri, n* = 22, 24. Bars represent mean +/− SEM. An unpaired two-sided Student's *t*-test was used to determine statistical significance between *trc Ri + con Ri* control and *trc Ri* + *additional RNAi* experimental genotypes, ** *P*-value <0.01, *** *P*-value <0.001. (C–E’’) Confocal images of terminal cells from third instar larvae (dorsal view) expressing *drm > GFP* (fluorescent signal) and the indicated RNAi transgenes. Bright field shows outline of lumen. Dashed square box indicates region that is zoomed in on the two right panels. Scale bars, 50 µM. Co-expression of *trc RNAi* with *fry RNAi* (C), *Mob2 RNAi* (D) or *Mo25 RNAi* (E) enhanced dilation rate in terminal cell transition zone (asterisk) compared with *trc RNAi* alone (Fig. 2b).

The RNAi lines were crossed to *drm > trc* RNAi and dorsal branch terminal cells were assessed at the third instar larval stage. Based on the outcomes of these interaction tests, candidate genes were categorized into 5 different classes: enhancers; suppressors; little or no effect; severe defects (where intact terminal cells were unable to be assessed due to disrupted tube morphology); and lethal (summarized in Fig. 2a). We observed that 24% of the RNAi lines screened enhanced the *drm > trc* RNAi dilation rate, where the top enhancers were RNAi lines against *furry* (*fry*) and glycolysis enzyme *Pyruvate Kinase* (*PyK*) (Supplementary Table 2). Eleven percent of RNAi lines screened suppressed the *trc* RNAi phenotype, where the strongest suppressor was an RNAi line against *Elongation factor 2* (*EF2*) (details in Supplementary Table 2). Other phenotypes were observed, such as gas-filling defects (where lumens in the terminal cell were not filled with gas) or gaps (where the tube was discontinuous). Where possible, multiple RNAi lines for each candidate gene were tested, with a total of 40 RNAi lines assessed overall. In some instances, RNAi lines against the same candidate genes produced inconsistent results (Supplementary Table 2). Not all terminal cells were scored for dilations due to tube morphology defects (such as gas-filling defects or gaps) in the transition zone, developmental delay or lethality.

A parallel RNAi screen was performed in a wild type background using a strong pan-tracheal driver *breathless-GAL4* with *UAS-Moesin::GFP* to mark tracheal tubes (*btl > MoeGFP*), to assess if knockdown of the selected Trc-binding proteins could phenocopy the dilation phenotype associated with depletion of GckIII pathway kinases (Supplementary Table 3). Other phenotypes such as wavy lumens or gaps were observed in terminal cells (Supplementary Table 3). Upon depletion of *PyK* or *Mob2* using RNAi, we observed a weak dilation phenotype in terminal cells (summarized in Supplementary Fig. 1, details in Supplementary Table 3), and other phenotypes such as gas-filling defects or gaps were also observed upon loss of *PyK* or *Mob2* in tracheal development (Supplementary Table 3).

Altogether, our screens identified numerous genes that, when depleted by themselves or in combination with *trc*, resulted in transition zone tube dilation. Of interest, RNAi against NDR scaffold proteins Furry and Mob2 and the glycolysis enzyme PyK were identified in both screens and were further assessed in tracheal development.

### Trc genetically interacts with known GckIII and NDR scaffold proteins in trachea

Our finding that *fry* could genetically interact with *trc* in terminal cells suggested the possibility that other NDR scaffold proteins could play a role in tracheal development. Further, we observed a weak dilation phenotype with loss of NDR scaffold Mob2 in wild type terminal cells and, additionally, almost all of the remaining *Drosophila* orthologues of scaffold protein families that regulate GckIII and NDR kinases were identified in the Trc-GFP pulldown (refer to Supplementary Table 1), including Mo25, and Mob family proteins, Mats and Mob4 (but not Mob3). To assess whether these scaffold proteins are important in tracheal development, RNAi lines against Fry, Mo25 and all Mob family proteins were crossed to *drm > trc RNAi*, and the dorsal terminal cells of third instar larvae were assessed. Of these scaffold proteins tested, we observed that co-expression of RNAi lines against *fry* (Fig. 2c) or *Mo25* (Fig. 2e) with *drm > trc* RNAi enhanced the dilation rate to 68 and 55%, respectively (Fig. 2b). Furthermore, RNAi lines against the four *Drosophila* Mob family proteins were assessed (data not shown), and only *Mob2 RNAi* co-expression modified the *drm > trc* RNAi dilation phenotype resulting in 46% terminal cells harboring dilations (Fig. 2b and 2d). The observation that expression of RNAi transgenes against *fry*, *Mob2*, or *Mo25* alone, driven by *drm > GFP,* resulted in dilation rates of 20%, 10.8% or 0%, respectively, in terminal cells (Supplementary Fig. 2), suggested that the genetic interactions observed between *trc* and *fry*, *Mob2* or *Mo25* are likely to be synergistic.

In summary, for the first time in tracheal morphogenesis, we observed genetic interactions between *trc* with genes encoding GckIII scaffold protein Mo25, and NDR kinase scaffold proteins Fry and Mob2. This is in keeping with the conserved function of these scaffold proteins in controlling Hippo-like signaling modules in different biological contexts and organisms (Maerz and Seiler 2010; Duhart and Raftery 2020).

### *Trc* genetically interacts with *PyK* in trachea

**Fig. 3.**
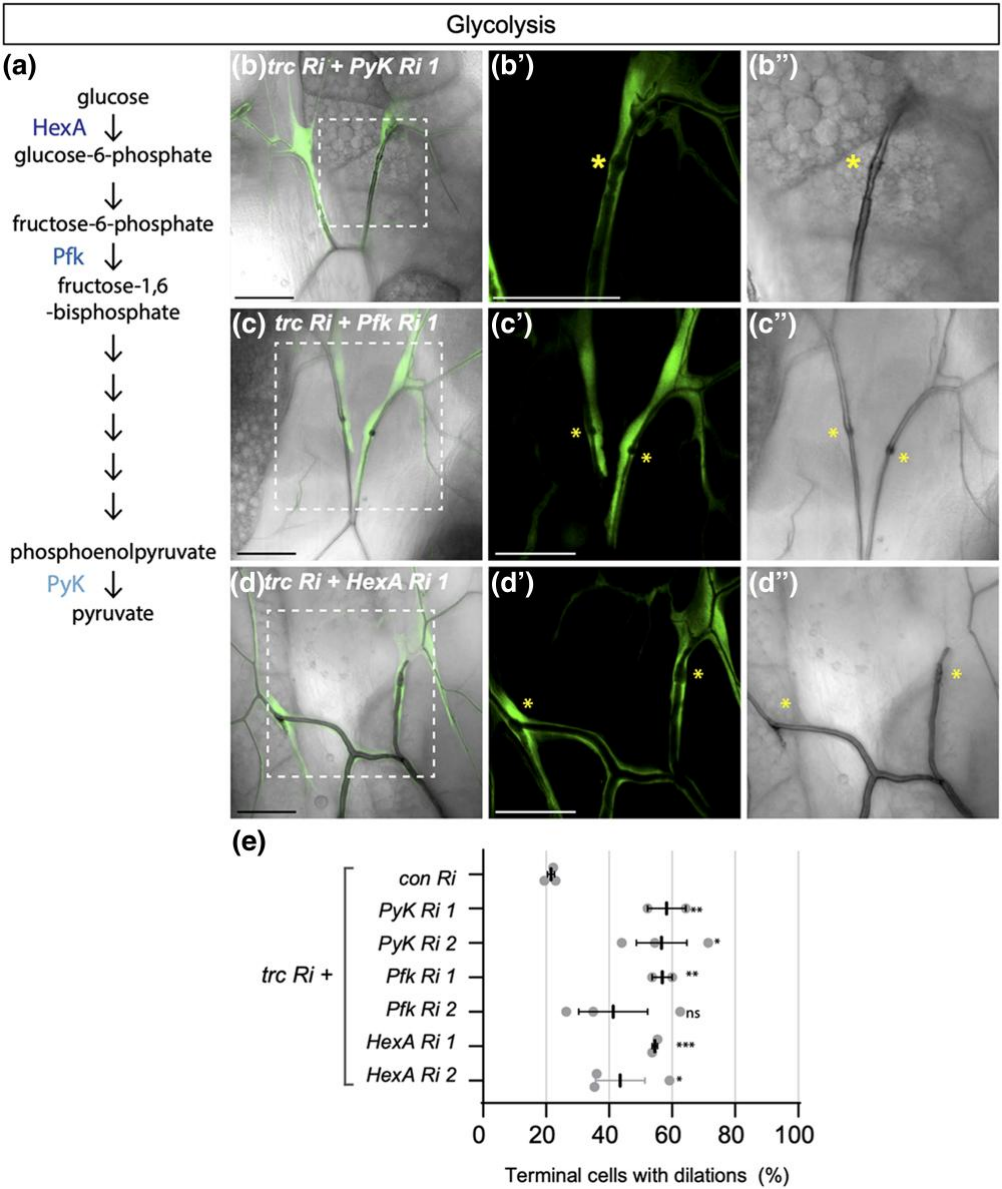
Loss of glycolysis enzymes enhances *trc* in terminal cells. a) Schematic of glycolysis, indicating hexokinase (HexA), phosphofructokinase (Pfk) and Pyruvate Kinase (PyK) enzymes. (B-D) Confocal images of terminal cells from third instar larvae (dorsal view) expressing *drm > GFP* (fluorescent signal) and the indicated RNAi transgenes. Bright field shows outline of lumen. Dashed square box indicates region that is zoomed in on the two right panels. Scale bars, 50 µM. Co-expression of *trc RNAi* with *PyK RNAi 1* (B-B’’), *Pfk RNAi 1* (C–C’’) or *HexA RNAi 1* (D–E’’) enhanced dilation rate in terminal cell transition zone (asterisk) compared with *trc RNAi* alone (Fig. 1d). e) Quantification of the number of terminal cells with dilations. Indicated RNAi lines are expressed by *drm > GFP*. Mean +/− SEM shown. Genotypes and number of terminal cells assessed (*n* value): *trc Ri* + *control Ri, n* = 27, 36, 26; *trc Ri* + *Pyk Ri 1, n* = 26, 29, 23; *trc Ri* + *Pyk Ri 2*, *n* = 47, 37, 56; *trc Ri* + *Pfk Ri 1, n* = 30, 82; *trc Ri* + *Pfk Ri 2, n* = 53, 43, 40; *trc Ri* + *HexA Ri 1, n* = 38, 69; *trc Ri* + *HexA Ri 2, n* = 71, 65, 50. Bars represent mean +/− SEM. An unpaired two-sided Student's t-test was used to determine statistical significance between *trc Ri + con Ri* control and *trc Ri* + *additional RNAi* experimental genotypes, * *P*-value <0.05, ** *P*-value <0.01.

PyK is a rate-limiting enzyme at the final stage of glycolysis that catalyzes the production of pyruvate and ATP to power cellular energy requirements (Fig. 3a) (Olson *et al*. 2016). For 2 of 3 RNAi lines assessed, knock down of *PyK* consistently enhanced the *drm > trc* RNAi dilation phenotype (50.3 and 55.1%, respectively; Fig. 3b, Supplementary Figure S3A, quantified in Fig. 3e). Knockdown of Pyk by itself, using the *drm-GAL4* driver, did not significantly affect terminal cell morphology (data not shown), but when knock down was carried out with a stronger tracheal *GAL4* driver (*btl > MoeGFP*), a weak dilation phenotype was observed. That depletion of *PyK* can both enhance the *drm > trc* RNAi phenotype, and at higher levels of knockdown, independently induce transition zone dilations, suggests that glycolysis plays an unanticipated role in tube morphogenesis.

The finding that *trc* genetically interacts with *PyK* in tracheal terminal cells suggested that cellular metabolism may regulate, or be required for, the same steps of tube morphogenesis controlled by the GckIII-Trc pathway. To test whether the results indicate a novel function of PyK, or whether glycolysis itself is linked to tube morphogenesis, we investigated if other key glycolytic enzymes could similarly interact with *trc*.

### Genetic interactions between trc and cellular metabolism in trachea development

To further assess the potential link between Trc and glycolysis, RNAi lines targeting two upstream glycolytic enzymes, Hexokinase A (HexA) and Phosphofructokinase (Pfk, Fig. 3a schematic), were tested for their ability to enhance *drm > trc* RNAi. *Pfk* RNAi lines enhanced *drm > trc* RNAi dilation rates to 57 and 40%, respectively (Fig. 3c and 3e, Supplementary Fig. 3b). Of interest, Pfk was also identified as a Trc-binding protein (Supplementary Table 1). Likewise, two *HexA RNAi* lines enhanced *drm > trc* RNAi dilation rates to 57 and 44%, respectively (Fig. 3d and 3e, Supplementary Fig. 3c). Expression of *Pfk* RNAi or *HexA* RNAi lines alone with *drm > GFP* resulted in a small number of dilations (Supplementary Fig. 3d). We also tested whether Lactate dehydrogenase (Ldh), a key enzyme in anaerobic phase of glycolysis would show genetic interaction with *trc*; however, we observed no significant effect on the *drm > trc* RNAi dilation rate (Supplementary Figure 3E). In sum, knockdown of *HexA, Pfk* and *PyK* all enhanced the frequency of dilations in *drm > trc RNAi* terminal cells, indicating a potential involvement of glycolysis as part of the GckIII-Trc signaling pathway.

####  

##### Trc RNAi dilation phenotype is enhanced upon disruption of oxidative phosphorylation

**Fig. 4.**
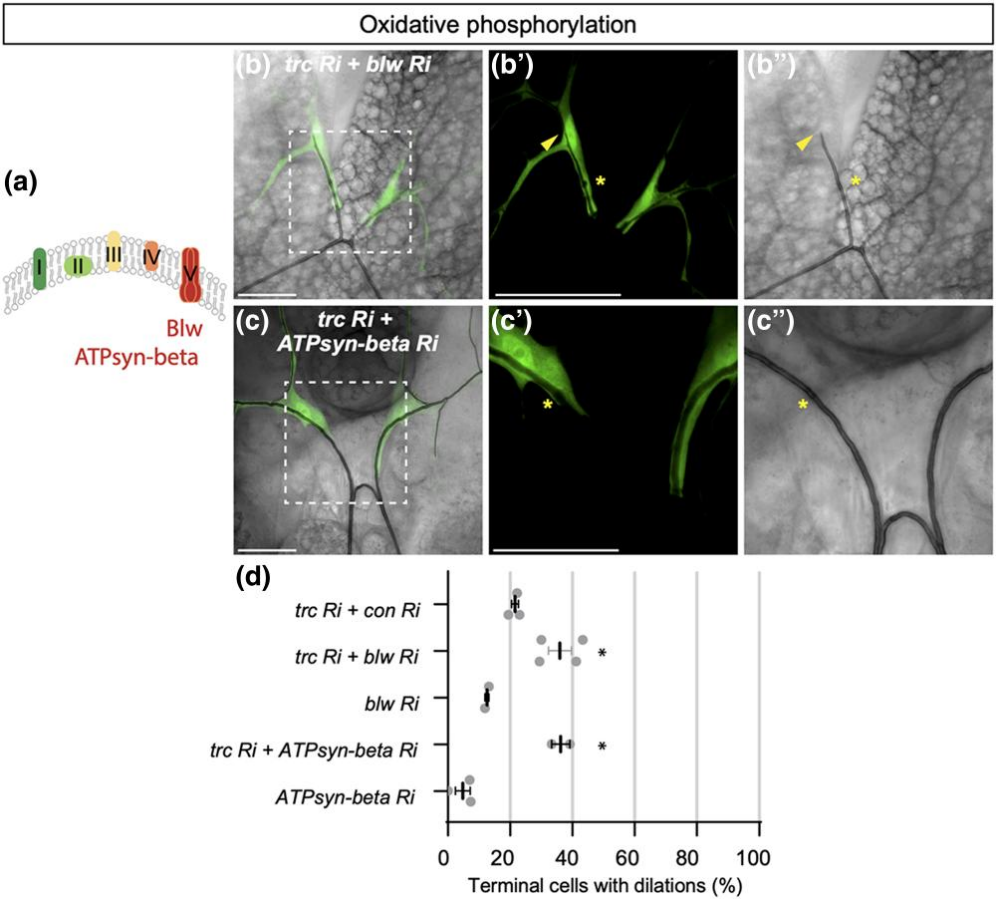
*trc* dilation phenotype is enhanced upon loss of oxidation phosphorylation in terminal cells. a) Schematic of electron transport chain complexes I-V. *bellwether* (blw) and *ATPsynthase-beta* (*ATPsyn-beta*) encode subunits of ATP synthase in complex V. (B-C’’) Confocal images of terminal cells from third instar larvae (dorsal view) expressing *drm > GFP* (fluorescent signal) and the indicated RNAi transgenes. Bright field shows outline of lumen. The dashed square box indicates the zoomed in region on the two panels on the right. Scale bars, 50 µM. Co-expression of *trc RNAi* using *drm > GFP* with *blw RNAi* (B-B’’) or *ATPsyn-beta RNAi* (C-C’’) enhances the dilation rate in terminal cell transition zone compared with *trc RNAi* alone (D). d) Quantification of the number of terminal cells with dilations. Indicated RNAi lines are expressed by *drm > GFP*. Mean +/− SEM shown. Genotypes and number of terminal cells assessed (*n* value): *trc Ri* + *control Ri, n* = 27, 36, 26; *trc Ri* + *blw Ri, n* = 30, 17, 17, 20; *blw Ri, n* = 59, 53; *trc Ri* + *ATPsyn-beta Ri, n* = 27, 23; *ATPsyn-beta Ri, n* = 41, 20, 43. Bars represent mean +/− SEM. An unpaired two-sided Student's t-test was used to determine statistical significance between *trc Ri + con Ri* control and *trc Ri* + *additional RNAi* experimental genotypes, ** *P*-value <0.01, * *P*-value < 0.05. e) We propose a model where CCM3 functions through the GckIII-NDR pathway kinase cascade to maintain correct tracheal morphogenesis. Known NDR kinase scaffold proteins Furry and/or Mob2 are likely to act with Trc in tracheal development.

Having found that knockdown of rate-limiting enzymes involved in glycolysis could enhance the *trc RNAi* dilation phenotype, we tested whether knockdown of oxidative phosphorylation enzymes, predicted to more severely affect ATP production, would also modify the *trc RNAi* in trachea development. Oxidative phosphorylation takes place in the mitochondria in five protein complexes that form the respiratory chain within the inner membrane (schematic in Fig. 4a). Interestingly, components of these complexes were identified as Trc-binding proteins in the Trc-GFP pulldown, such as complex I component NADH dehydrogenase NADH dehydrogenase (ubiquinone) 75 kDa (ND75), a core subunit of the NADH dehydrogenase, and complex V components Bellwether (Blw, the ATP synthase alpha subunit) and ATP synthase-beta (ATPsyn-beta, the ATP synthase-beta subunit) (Supplementary Table 1). We used validated RNAi lines (Van Den Ameele and Brand 2019) to knock down these putative Trc-interactors and assess their ability to modify the *drm > trc* RNAi phenotype in terminal cells.

NADH hydrogenase is critical for transferring electrons from NADH to the respiratory chain. We found that knockdown of subunit ND75 using 2 independent RNAi lines in *drm > trc RNAi* terminal cells did result in a small increase in the dilation rate of 26.9 and 33.6%, respectively, however, this effect was not significant compared to control RNAi (Supplementary Fig. 3f–h). Expression of *ND75* RNAi line 1 or 2 alone with *drm > GFP* caused dilations in 8.5 and 4.7% of terminal cells, respectively (Supplementary Fig. 3h). In addition, a significant number of terminal cells exhibited gas-filling defects in terminal cell lumens when co-expressing *trc* RNAi with either *ND75* RNAi line 1 (70.0%) or *ND75* RNAi line 2 (43.4%, data not shown).

ATPsynthase is composed of multiple subunits and functions in the last step of oxidative phosphorylation (Pena and Garesse 1993; Boyer 1997). Knockdown of the ATP synthase alpha subunit, encoded by *blw*, enhanced the *drm > trc RNAi* dilation rate to 35.9% compared with a control RNAi (Fig. 4b and 4d). Similarly, RNAi knockdown of *ATPsyn-beta* catalytic subunit enhanced the *drm > trc* RNAi dilation rate to 36.2% (Fig. 4c and 4d). Furthermore, knockdown of either *blw* or *ATPsyn*-*beta* in otherwise wild type terminal cells resulted in dilation rates of 12.5 and 4.8%, respectively (Fig. 4d), suggesting (1) that the interaction between *trc RNAi* and knockdown of ATPsynthase subunits tested are synergistic and 2) that functional ATPsynthase itself may be required to maintain tube morphology in homeostasis.

In addition, upon inhibition of oxidative when expressing RNAi against *Blw1*, *ATPsyn-beta* or *ND75* in wild type terminal cells, we observed considerable disruption to the lumens, notably an increase in the number of terminal cells with gas-filling defects (between a range of ∼20–50%, Supplementary Fig. 4b). The gas-filling defect phenotype was enhanced when the RNAi lines against *Blw1*, *ATPsyn-beta* or *ND75* were each co-expressed with *trc* RNAi, resulting in an increased number of terminal cells exhibiting gas-filling defects (between a range of 40–70%, Supplementary Fig. 4b) that was statistically significant compared with expression of *trc* RNAi with a control RNAi. The effect of disrupting mitochondrial function on gas-filling of trachea has been previously reported (Papakyrikos *et al.* 2020), and thus is expected. That knockdown of *trc* and mitochondrial gene function are mutually enhancing (for tube dilation and gas-filling) is striking.

Due to the disruption to some terminal cell tube morphology, it is difficult to discern the true effect of knockdown of complex I or V components on the *trc* RNAi dilation rate under current experimental conditions. Further investigation, such as using clonal analysis to mitigate overall disruption to tracheal architecture, will be required to determine the role of oxidative phosphorylation in trachea and better assess the genetic interaction with *trc* RNAi in terminal cells. Altogether, these data suggest that *trc* may integrate changes in cellular metabolism with tubule growth and morphology.

## Discussion

To investigate GckIII pathway signaling in trachea, we performed biochemical and genetic screens to identify effectors of NDR kinase Trc, the most downstream protein characterized in the GckIII pathway thus far. Despite scrutiny of Hippo-like signaling pathway cascades in different organisms, few NDR substrates have been identified and fully characterized (Maerz and Seiler 2010; Hergovich 2016). Here, we report two key findings that inform how GckIII pathway signaling might be regulated in tracheal development and suggest its potential biological impact. First, we show that *trc* genetically interacts with *Ccm3* suggesting a role for a GCKIII-NDR kinase module downstream of CCM signaling in tracheal development, and perhaps more broadly in other contexts. In humans, mutation of *Ccm3* leads to an autosomal dominant vascular disease in which brain capillaries become grossly dilated and leaky. Three human genes have been implicated in the disease, and their protein products are thought to act together as a ternary complex, signaling through a MEKK3-MEK5-ERK5 cascade to regulate KLF2/4 (reviewed in Su and Calderwood 2020). However, conflicting data raise the possibility that CCM3 may act through a distinct pathway from its binding partners CCM1/KRIT1 and CCM2/MALCAVERNIN, and the molecular connections between the CCM1/2/3 complex and the presumed downstream effectors KLF2/4 remain unknown. The genetic interaction we observe between *ccm3* and *trc*, strengthens the model that CCM3 is part of a Hippo-like signaling cassette that exerts its influence on morphogenesis through a GCKIII-TRC/NDR kinase cascade.

Second, we uncover multiple genetic interactions between trc and components of the ATP generating glycolysis and oxidative phosphorylation pathways. This suggests an unanticipated connection between changes in cellular energy, as controlled by glycolysis and mitochondrial respiration, and signaling though the GckIII pathway to support tube morphogenesis in tracheal terminal cells. This connection may run through converging independent pathways, or may imply a regulatory relationship between the two.

### A potential function for hippo-like pathway signaling downstream of CCM3

We identified genetic interactions between *trc* and two proteins associated with GckIII kinases in different organisms: *Ccm3* and *Mo25* (Nozaki *et al.* 1996; Ma *et al.* 2007; Fidalgo *et al.* 2010; Zheng *et al.* 2010). In humans, three unrelated genes have been identified that, when mutant, cause autosomal dominant familial vascular disease. Although the gene products of the three loci form a ternary complex and have been thought to act together to regulate downstream targets, the loss of CCM3 is associated with the most severe prognosis, and is associated with unique defects as compared with CCM1 and CCM2 (Lant *et al.* 2015, 2018; Retta *et al.* 2020). Dimerization of CCM3 with a GCKIII kinase binding partner is well established (Fidalgo *et al.* 2010; Ceccarelli *et al.* 2011; Zhang *et al.* 2013); however, the effector signals downstream of GCKIII kinases and CCM3 proteins have been less clear. Indeed, the molecular connections between the CCM proteins and the known downstream effectors in the vascular system are unknown. Our finding that *Ccm3* genetically interacts with *trc* in trachea development suggests that a Hippo-like GCKIII-NDR signaling module could act downstream of CCMs in tubulogenesis, with potential relevance to CCM biology in vascular disease.

Mo25 is an armadillo-repeat protein that is typically linked to the Germinal center kinase (Gck) subfamily in fungi and human cells (Dettmann *et al.* 2012; Fuller *et al.* 2012; Shi *et al.* 2013; Bizotto *et al.* 2018). In fungi, Mo25 facilitates GckIII-NDR kinase driven morphogenesis programs, such as regulation of Ace2p transcription factor and polarized morphogenesis or RAM (Maerz and Seiler 2010; Bizotto *et al.* 2018). A ternary complex between Mo25-GckIII-CCM3 has been described in the *C. elegans* excretory canal, also composed of seamless tubes, where Mo25 controls canal integrity by regulating CCM3-GCKIII localization (Lant *et al.* 2018). Based on our observations and findings in previous studies, it will be of interest to assess whether Mo25 regulates GckIII-CCM3 localization or if it acts as a linker between GckIII and Trc kinases to facilitate their binding and/or activation in trachea development, and whether these interactions are conserved in vertebrate CCM models. Furthermore, it will be important to determine whether GCKIII-NDR kinase signaling functions downstream of CCM3 in the vertebrate vascular system, and how it interacts with other CCM effectors such as Rho (Zheng *et al.* 2010) in order to understand how cerebral capillary morphology is regulated, and how it goes awry in human disease.

### A role for conserved regulators of hippo-like signaling modules in trachea development

Scaffold proteins are important regulators of GckIII and NDR kinases in a variety of contexts and model organisms (Thompson and Sahai 2015). In regulating NDR kinase function, Fry and Mob family proteins are key partners in many settings. Fry was the highest ranking scaffold protein identified as a Trc interactor, consistent with previous studies in fly and human cultured cells demonstrating Fry and Trc-binding (Chiba *et al.* 2009; Fang and Adler 2010; Fang *et al.* 2010). We found that *fry* and *trc* also genetically interact in the context of trachea development, and predict a positive relationship between Fry and Trc, akin to that observed in other settings in *Drosophila*, such as in sensory neurons (Emoto *et al.* 2004) or wing bristles (Cong *et al.* 2001; He *et al.* 2005a; Fang *et al.* 2010). Indeed, subsequent study of EMS-induced *fry* mutations in *Drosophila* tracheal terminal cells demonstrate a clear phenocopy of the *GckIII* mutations (Antwi-Adjei 2021).

Of the four Mob family proteins in *Drosophila*, only *Mob2* genetically interacted with *trc* in our terminal cell assays. Biochemical data clearly shows that Mob2 and Trc form a stable complex in fly and human cells (Devroe *et al.* 2004; He *et al*. 2005; Gundogdu and Hergovich 2019), and in flies, genetic experiments suggest that Mob2 activates Trc in wing bristle morphogenesis (He *et al*. 2005). Our genetic interaction data support a scenario where Mob2 and Trc may function in a common pathway in tracheal development.

The fact that Trc acts with multiple scaffold proteins in terminal cells is consistent with studies in wing bristles, where the *trc* mutant multiple wing hair phenotype is enhanced by loss of either *fry* or *Mob2* (He *et al*. 2005). However, in contrast with our findings in trachea terminal cells here, *trc* also genetically interacts with another Mob family member *mats,* but not *Mo25* in wing bristles (He *et al*. 2005). Precisely why, when and how these proteins coordinate Trc activity in different settings is not fully understood and the precise manner in which these multiple scaffold proteins control GckIII pathway signaling is likely to depend on cell type and/or developmental context.

### Coordinating GckIII pathway and metabolism to maintain tube morphology in tracheal development

That *trc* could genetically interact with metabolic enzymes is particularly interesting. While metabolic enzymes are unquestionably essential for generating energy and building blocks for cellular processes, they can also provide instructive signals, for example in patterning and differentiation programs (Teixeira *et al.* 2015; Lu and Hunter 2018; Miyazawa and Aulehla 2018; Genovese *et al.* 2019; Van Den Ameele and Brand 2019). Our findings suggest that cellular metabolism may be important to support changes in tube morphology and raises the possibility that GckIII pathway signaling could regulate or coordinate this response to maintain tube integrity in trachea morphogenesis. Cross-talk between metabolism, particularly glycolysis, and cell signaling pathways such as PI3K, VEGF, FGF, or Notch is well studied in vertebrate endothelial cells that form branched vascular systems comparable with tracheal architecture (Ochoa-espinosa *et al.* 2012; Uebelhoer and Iruela-Arispe 2016).

One recent report shows that pyruvate kinase muscle 2 (PKM2) isoform maintains ATP levels in order to stabilize endothelial cell junction dynamics and remodeling, independent of cell proliferation (Gómez-Escudero *et al.* 2019). The requirement of glycolysis to regulate cell junction stability of endothelial cells may be applicable to the junction remodeling process that occurs in terminal cell transition zones, a region that appears sensitive to deficiencies in GckIII pathway kinases and where septate junction and apical proteins are aberrantly localized (Song *et al*. 2013; Poon *et al.* 2018). In future studies, it will be crucial to determine if ATP levels are affected by loss of *PyK* or *trc* in *Drosophila* terminal cells—for example, by using ATP sensors (Tsuyama *et al.* 2013). Likewise, it will be important to determine whether changes in ATP production correlate with the extent of junctional remodeling in the transition zone where proper connection of autocellular to seamless tubes is critical.

It is currently unclear what the regulatory relationship between Trc and PyK might be, and whether Trc and PyK function in a common pathway or in parallel with GckIII pathway signaling to regulate trachea. Further biochemical studies, such as assessing whether Trc and PyK directly bind, in combination with genetic epistasis experiments to determine the genetic hierarchy between PyK and Trc will be required to elucidate the regulatory relationship between Trc and PyK, and perhaps other glycolysis enzymes such as HexA and Pfk.

### GckIII pathway kinases in cellular metabolism

The suggestion that Trc might control or coordinate cellular energy is tantalizing; how and where might Trc interact with metabolic enzymes? In *Drosophila*, the most established role for Trc is in polarized epidermal extensions, including bristles in the developing and adult wing. In the wing, Trc is suggested to function via effects on the actin cytoskeleton, although the underlying molecular mechanism is as yet unclear (Geng *et al.* 2000; Cong *et al.* 2001; He *et al*. 2005; Fang and Adler 2010). Of note, in vertebrate endothelial cells, metabolic enzymes such as phosphofructokinase and pyruvate kinase colocalize with F-actin and are enriched in membrane ruffles at the leading front of lamellipodia during sprouting (De Bock *et al.* 2013). The proposed compartmentalization of glycolytic enzymes with F-actin could be advantageous for many reasons, including containing ATP supply to a defined region and enabling proximity of glycolytic enzymes to potential activators or regulators, such as Trc, if a rapid response is required. Indeed, in the fly, Trc partially colocalises with F-actin in the wing bristle, forming puncta along developing these epidermal extensions (He *et al.* 2005).

Another cellular compartment where Trc might potentially influence cellular energy is at the mitochondria itself. In muscle cells, mitochondrial kinase PTEN-induced kinase 1 (PINK1) preserves the integrity and function of mitochondria in the conserved process of mitochondrial quality control and Trc activation is proposed to be partly dependent on PINK1 in this process (Wu *et al.* 2013). Significantly, activated Trc was associated with mitochondrial fractions of muscle cells, suggesting the mitochondria as another setting where active Trc functions. Intriguingly, Hippo pathway regulator Fat/FAT4 has also been linked to mitochondrial regulation of oxidative phosphorylation, with a fragment of Fat found to interact with ND24 (a NAD dehydrogenase subunit) and Belweather the α subunit of ATPsyn (Sing *et al.* 2014).

Although GckIII kinase is yet to be connected to metabolism in *Drosophila*, vertebrate GCKIII family kinases (MST3, MST4, and STK25) are associated with metabolic functions. For example, STK25 is important in lipid metabolism associated with liver, muscle, and adipose tissues, and MST3 is associated with glucose metabolism in the liver or glucose and insulin homeostasis in a Type 2 diabetes mouse model (Iglesias *et al.* 2017; Wu *et al.* 2018; Cansby *et al.* 2019; Pombo *et al.* 2019). In cultured human cell lines, all three vertebrate GCKIII kinases are activated in response to oxidative stress (Pombo *et al.* 1996; Wu *et al.* 2011; Fidalgo *et al.* 2012). In colorectal cancer cell lines, concomitant with repressing cell proliferation, STK25 inhibits glycolysis by reducing expression of glycolysis enzymes, such as Glucose transporter 1 (GLUT1), Hexokinase 2 (HK2), Pyruvate kinase isozyme M2 (PKM2), lactate dehydrogenase A (LDHA), and pyruvate dehydrogenase 1 (PDHK1), at the mRNA and protein level (Wu *et al.* 2018). Here, the authors show that STK25 binds to Golgi protein GOLPH3, which is proposed to activate mTOR signaling to indirectly control glycolysis. It is unclear whether an equivalent mode of transcriptional regulation of glycolysis enzymes could apply to our understanding of GckIII pathway function in the largely non-mitotic cells of the larval trachea network. One common thread is that GckIII kinases respond to dietary changes that affect glucose or lipid metabolism. One recent study has shown that nutrition can promote tracheal branching and increased tracheal area around developing brain hemispheres (Yuan *et al.* 2020). It may be worth investigating if modifying diet could influence morphogenesis of the seamless tube itself and whether this adaptability is driven by GckIII pathway signaling.

Here we adopted biochemical and genetic approaches to investigate the GckIII pathway in trachea development. We observed for the first time an in vivo genetic interaction between Trc and GckIII scaffold protein CCM3 in trachea development. Along with our previous findings (Poon *et al*. 2018), we propose that CCM3 may function through the GckIII-NDR pathway kinase cascade to influence tracheal morphogenesis in *Drosophila* (please refer to the model in Fig. 4e). Furthermore, our results suggest that scaffold proteins Furry and/or Mob2 may also act with Trc in tracheal development (Fig. 4e), suggesting a conserved role for these scaffold proteins in regulating NDR kinase function in different biological settings. Given the key role of CCM proteins in human vascular disease and the established role of GCKIII kinases as an effector of CCM proteins in CCM (Fischer *et al.* 2013), it will be key to determine if a Hippo-like GCKIII-NDR pathway functions downstream of CCM proteins in mammalian models and may provide opportunities for therapeutic intervention. In addition, our observation that Trc and metabolism genetically interact suggests a potential function of the GckIII pathway to mediate cellular energy required to preserve tube integrity and may represent an additional responsive mechanism utilized by trachea to maintain their fine branched architecture.

## Supplementary Material

jkad013_Supplementary_Data

## Data Availability

Fly strains and plasmids are available upon request. Mass spectrometry data is available from PRIDE (PXD038912). The authors affirm that all data necessary for confirming the conclusions of the article are present within the article, figures, and tables. Supplemental material available at G3 online.
