# Supplementary material for: NDR kinase tricornered genetically interacts with Ccm3 and metabolic enzymes in Drosophila melanogaster tracheal development
Source: G3 (Bethesda). 2023 Jan 19;13(3):jkad013. doi: 10.1093/g3journal/jkad013 (PMC9997570; doi:10.1093/g3journal/jkad013)
Supplement: jkad013_Supplementary_Data [file jkad013_supplementary_data.zip › Supplemental_Figure_Legends_G3-2022-403984.docx]

**SUPPLEMENTAL FIGURE LEGENDS**

**Supplemental Figure S1**

(A) Summary of phenotypic categories identified in *btl>MoeGFP* genetic *RNAi* screen.

(B) Terminal cells from third instar larvae (dorsal view) expressing *drm>GFP* (marked by green) and the indicated RNAi transgenes. Bright field shows outline of lumen. Expression of *PyK RNAi* (B-B’) or Mob2 RNAi (C-C’) with *btl>MoeGFP* resulted in dilations in terminal cells.

**Supplemental Figure S2**

(A-C) Confocal images of terminal cells from third instar larvae (dorsal view) expressing *drm>GFP* (marked by green) and the indicated RNAi transgenes. Bright field shows outline of lumen. Scale bars, 50 µM. Expression of *furry RNAi* (A), *Mob2 RNAi* (B) or *Mo25 RNAi* (C) using *drm>GFP* resulted in moderate effects on terminal cells, including wavy lumen phenotypes. Mean +/- SEM shown. (D) Quantification of the number of terminal cells with dilations. Indicated RNAi lines are expressed by *drm>GFP*. Mean +/- SEM shown. Genotypes and number of terminal cells assessed (n value): *fry Ri*, n = 17, 15, Mob2 Ri, n = 42, 22, 35, 64; *Mo25 Ri*, n = 22, 24. Bars represent mean +/- SEM.

**Supplemental Figure S3**

(A-C, F-G) Confocal images of terminal cells from third instar larvae (dorsal view) expressing *drm>GFP* (marked by green) and the indicated RNAi transgenes. Bright field shows outline of lumen. Asterisks mark dilations, arrowhead marks gaps in lumen and arrows marks gas filled defects in tubes. Scale bars, 50 µM. Co-expression of *trc RNAi* using *drm>GFP* with *PyK RNAi 2* (A-A’), *Pfk RNAi 2* (B-B’) or *HexA RNAi 2* (C-C’) enhanced dilation rate in terminal cell transition zone (asterisk) compared to *trc RNAi* alone (Figure 1D, refer to Figure 3E for quantification).

Co-expression of *trc RNAi* using *drm>GFP* with *ND75 RNAi 1* (F-F’) or *ND75 RNAi 2* (G-G’) did not significantly alter dilation rate compared control terminal cells expressing *trc RNAi* and *control (con, luciferase) RNAi* (quantified in H). Expression of *ND75 RNAi 1* or *ND75 RNAi 2* alone with *drm>GFP* causes dilations in 8.5% and 4.7%, respectively, of terminal cells assessed (data not shown, quantified in H). Quantification of the number of dilations in terminal cells expressing *drm>GFP* of the indicated genotypes. Genotypes and number of terminal cells assessed (n value): (D) *HexA* *Ri 1,* n = 19, 39; *HexA Ri 2, n = 39, 24; Pfk Ri 1, n = 32, 51;* (E) *trc Ri* + *control Ri,* n = 27, 36, 26; *trc Ri* + *Ldh Ri,* n = 36, 39, 61; *Ldh Ri, n = 36, 30 ;* (H) *trc Ri* + *control Ri,* n = 27, 36, 26; *trc Ri* + *ND75 Ri 1,* n = 40, 24, 50, 49; *ND75 Ri 1,* n = 39, 46, 45; *trc Ri* + *ND75 Ri 2*, n = 44, 44, 26; *ND75 Ri 2*, n = 50, 33, 48.Bars represent mean +/- SEM. An unpaired two-sided Student’s t-test was used to determine statistical significance between *trc Ri + con Ri* control and *trc Ri* + *additional RNAi* experimental genotypes, ns = not significant.

**Supplemental Figure S4**

(A-B) Quantification of the number of terminal cells of third instar larvae co-expressing *trc RNAi* and *RNAi* against genes involved in glycolysis (A) or oxidative phosphorylation (B) with gas-filling defects. Indicated RNAi lines are expressed by *drm>GFP*. . Genotypes and number of terminal cells assessed (n value): (A) *trc Ri* + *control Ri,* n = 27, 36, 26; *trc Ri* + *Pyk Ri 1,* n = 26, 29, 23; *trc Ri* + *Pyk Ri 2*, n = 47, 37, 56; *trc Ri* + *HexA* *Ri 1,* n = 38, 69; *trc Ri* + *HexA Ri 2,* n = 71, 65, 50; *trc Ri* + *Pfk Ri 1,* n = 30, 82; *trc Ri* + *Pfk Ri 2,* n = 53, 43, 40; (B) *trc Ri* + *control Ri,* n = 27, 36, 26; *trc Ri* + *blw Ri,* n =30, 17, 17, 20; *trc Ri* + *ATPsynbeta Ri,* n = 27, 23; *trc Ri* + *ND75 Ri 1,* n = 40, 24, 50, 49; ; *trc Ri* + *ND75 Ri 2*, n = 44, 44, 26; *blw Ri,* n = 59, 53; *ATPsynbeta Ri,* n = 41, 20, 43; *ND75 Ri 1,* n = 39, 46, 45 *ND75 Ri 2*, n = 50, 33, 48. Bars represent mean + SEM. An unpaired two-sided Student’s t-test was used to determine statistical significance between *trc Ri + con Ri* control and *trc Ri* + *additional RNAi* experimental genotypes, * p value < 0.05.
