# Supplementary figures and images for: NDR kinase tricornered genetically interacts with Ccm3 and metabolic enzymes in Drosophila melanogaster tracheal development
Source: G3 (Bethesda). 2023 Jan 19;13(3):jkad013. doi: 10.1093/g3journal/jkad013 (PMC9997570; doi:10.1093/g3journal/jkad013)

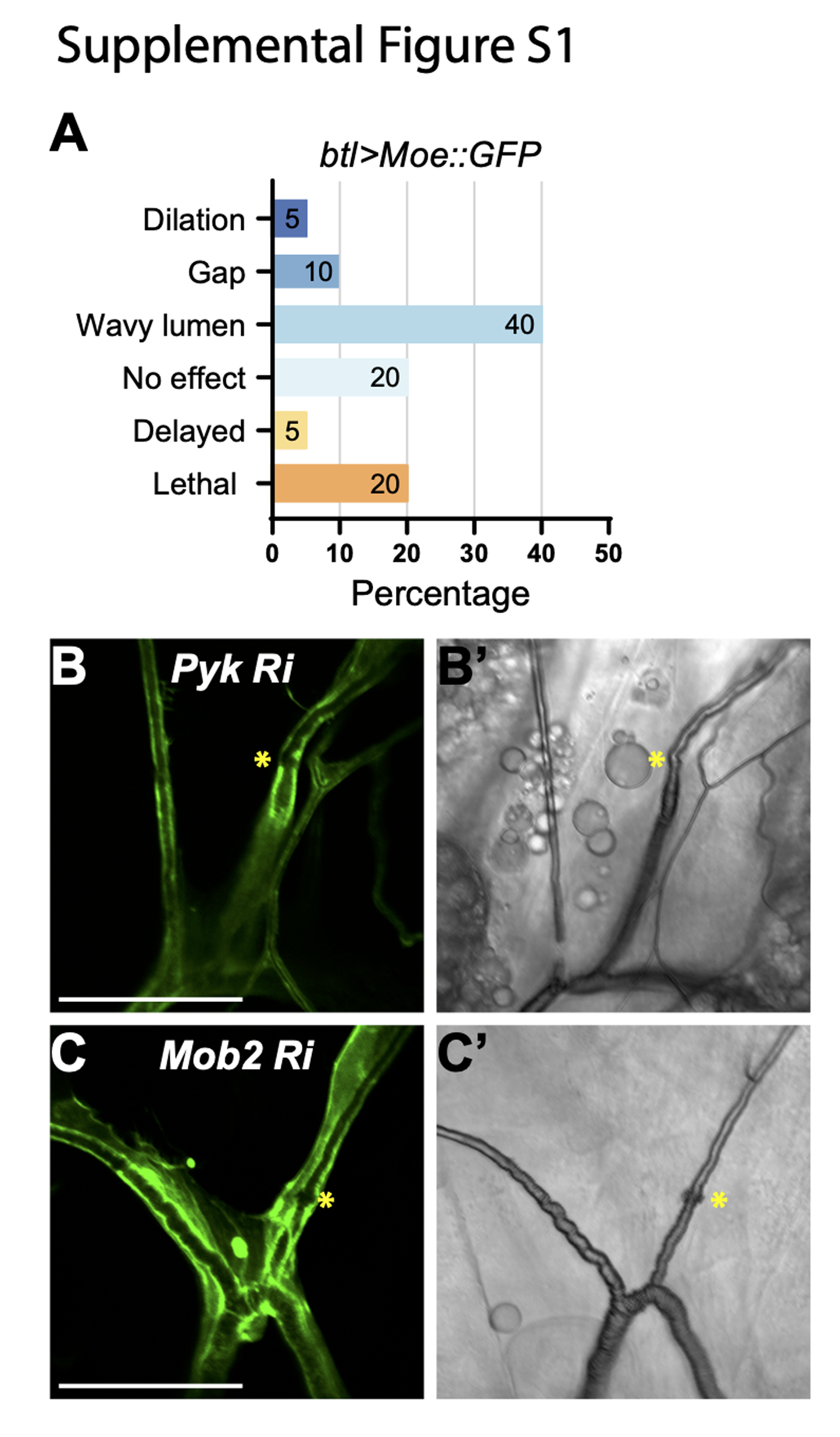

Supplement: jkad013_Supplementary_Data [file jkad013_supplementary_data.zip › Supplemental_Figure_S1_G3-2022-403984.tif]

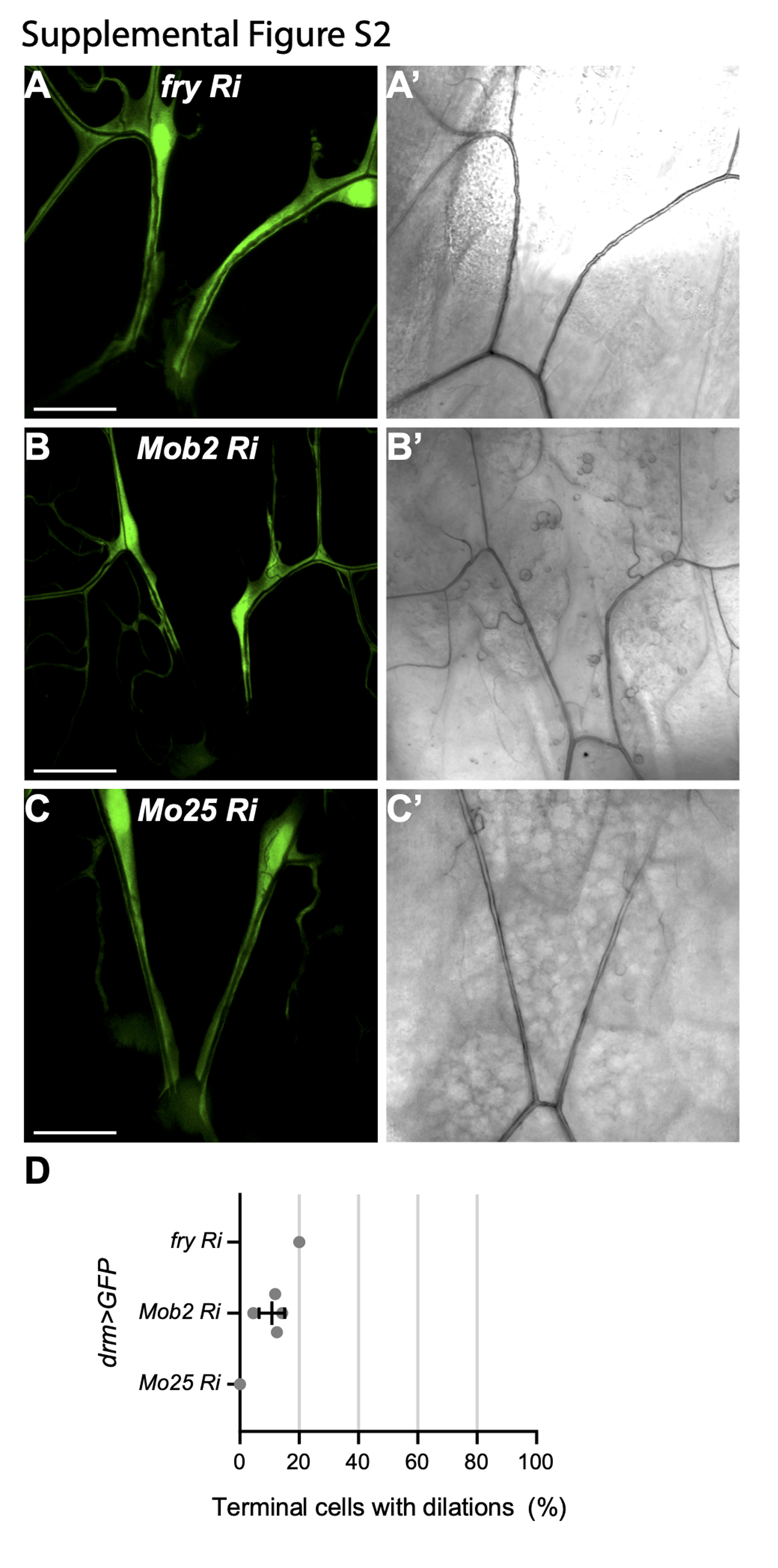

Supplement: jkad013_Supplementary_Data [file jkad013_supplementary_data.zip › Supplemental_Figure_S2_G3-2022-403984.tif]

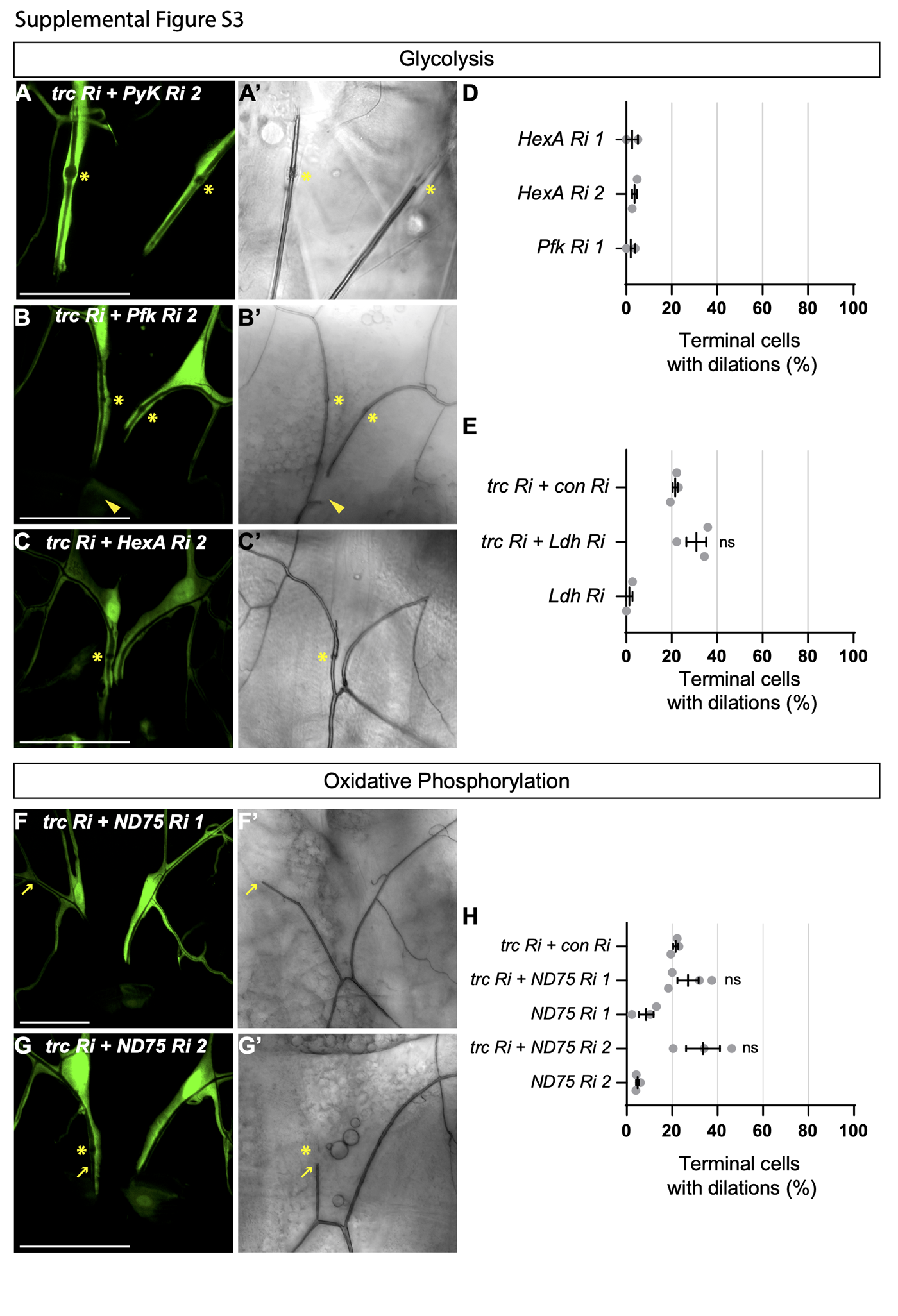

Supplement: jkad013_Supplementary_Data [file jkad013_supplementary_data.zip › Supplemental_Figure_S3_G3-2022-403984.tif]

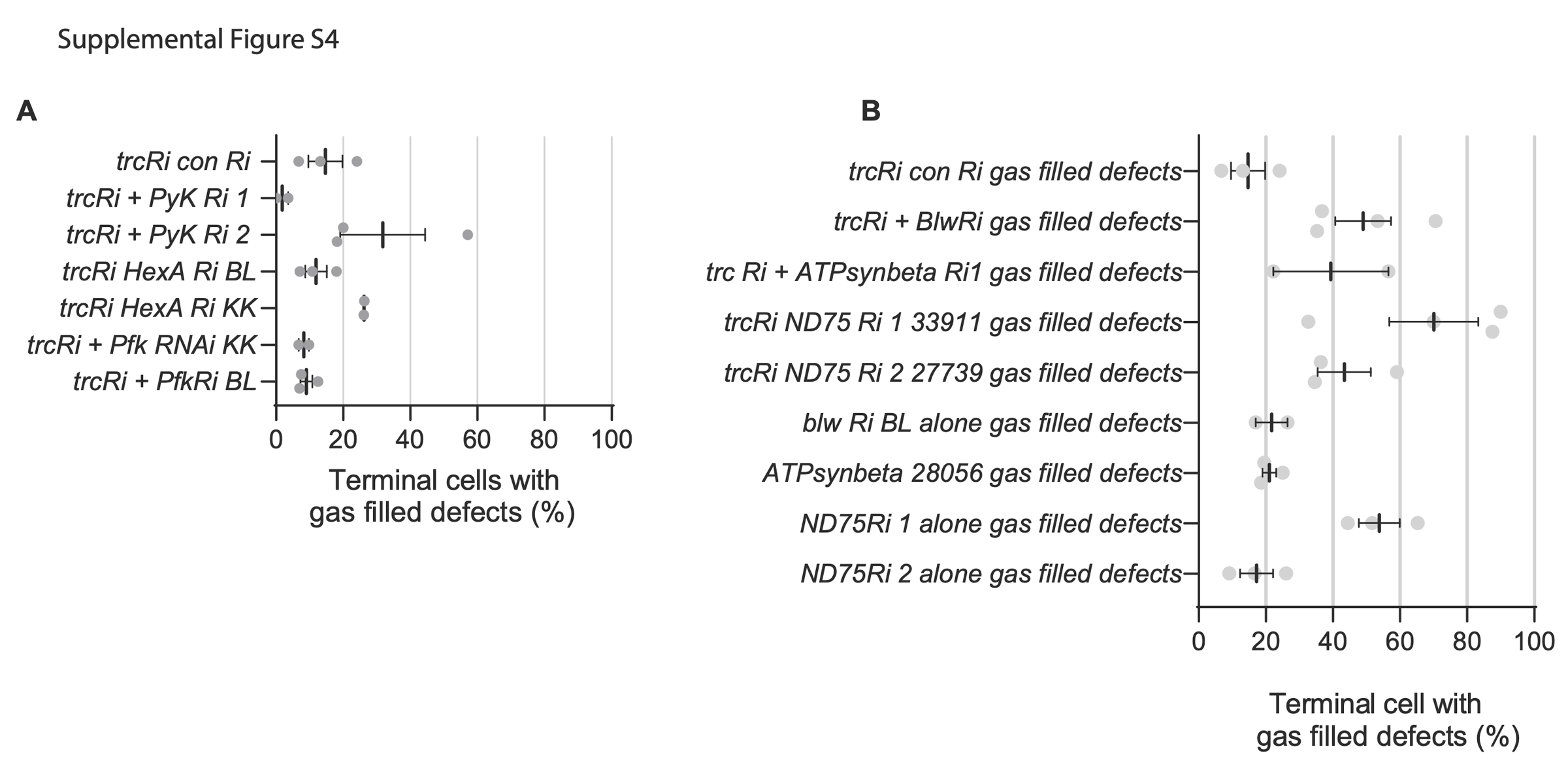

Supplement: jkad013_Supplementary_Data [file jkad013_supplementary_data.zip › Supplemental_Figure_S4_G3-2022-403984.tif]
